# A Conceptual Model of Nurses’ Turnover Intention

**DOI:** 10.3390/ijerph19138205

**Published:** 2022-07-05

**Authors:** Eva Smokrović, Tomislav Kizivat, Antun Bajan, Krešimir Šolić, Zvjezdana Gvozdanović, Nikolina Farčić, Boštjan Žvanut

**Affiliations:** 1 Faculty of Medicine, Josip Juraj Strossmayer University of Osijek, 31000 Osijek, Croatia; eva.smokrovic@uniri.hr (E.S.); tkizivat@mefos.hr (T.K.); antun.bajan@fdmz.hr (A.B.); kresimir@mefos.hr (K.Š.); 2 Faculty of Health Studies, University of Rijeka, 51000 Rijeka, Croatia; 3 Nursing Institute “Professor Radivoje Radić”, Faculty of Dental Medicine and Health Osijek, Josip Juraj Strossmayer University of Osijek, 31000 Osijek, Croatia; zvjezdana.gvozdanovic@obnasice.hr (Z.G.); nikolina.farcic@fdmz.hr (N.F.); 4 Faculty of Electrical Engineering, Computer Science and Information Technology, Josip Juraj Strossmayer University of Osijek, 31000 Osijek, Croatia; 5 Faculty of Health Sciences, University of Primorska, 6000 Koper, Slovenia

**Keywords:** turnover models, intrinsic motivation, turnover intention, nursing practice environment

## Abstract

The World Health Organisation predicts a lack of 15 million health professionals by 2030. The lack of licenced professionals is a problem that keeps emerging and is carefully studied on a global level. Strategic objectives aimed at stimulating employment, improving working conditions, and keeping the nurses on board greatly depends on identifying factors that contribute to their turnover. The aim of this study was to present a conceptual model based on predictors of nurses’ turnover intention. **Methods:** A quantitative, non-experimental research design was used. A total of 308 registered nurses (RNs) took part in the study. The Multidimensional Work Motivation Scale (MWMS) and Practice Environment Scale of the Nursing Work Index (PES-NWI) were used. **Results:** The conceptual model, based on the binary regression models, relies on two direct significant predictors and four indirect significant predictors of turnover intention. The direct predictors are job satisfaction (OR = 0.23) and absenteeism (OR = 2.5). Indirect predictors that affect turnover intention via job satisfaction are: amotivation (OR = 0.59), identified regulation (OR = 0.54), intrinsic motivation (OR = 1.67), and nurse manager ability, leadership and support of nurses (OR = 1.51). **Conclusions:** The results of the study indicate strategic issues that need to be addressed to retain the nursing workforce. There is a need to ensure positive perceptions and support from managers, maintain intrinsic motivation, and promote even higher levels of motivation to achieve satisfactory levels of job satisfaction.

## 1. Introduction

Since the early 1920s, the phenomenon of employee turnover has preoccupied many experts. With the advent of the 21st century, it became a global problem that spread to all areas of organisational processes. Considering the development of new strategies for employees’ retention, market dynamics, the development of research methodology, and technology, it is not surprising that employee turnover intention is a phenomenon that will be studied again and again. The practical application of theoretical knowledge and the development of conceptual models of employees’ turnover intention will help managers develop better and more efficient business practices that will ultimately optimise the use of organisational resources.

Turnover intention reflects the employees’ attitude towards the organisation, or, in other words, their conscious intention to leave the organisation [1]. An individual’s intention is identified as the most important cognitive antecedent of behaviour [2].

The healthcare sector is the largest group of employees in the world, and nurses account for the largest share in this group. The lack of licenced professionals is a recurring problem that is being carefully studied at the global level. Strategic objectives aimed at promoting employment, improving working conditions, and keeping nurses on board depend heavily on identifying the factors that contribute to their turnover. The results of numerous international studies indicate that there is a significant increase in the number of nurses who express their intention to change jobs [3,4].

Throughout history, various theories and models of turnover intention have been presented describing how to identify the key cognitive antecedent of this behaviour. Considering the presented constructs, in this study, we have singled out the ones that played an important role and thus presented a new model of nurses’ turnover intention.

### 1.1. Background

Even though the term turnover intention was not generally used until the middle of the 20th century, the first published work dates from 1925. It dealt with questions and answers about turnover intention among employees and it is considered as the antecedent of the standard model until the 1960s. Over the years that followed, scientists introduced methodological characteristics that are still used today in studies that look into employees’ attitudes, workplace conditions, job satisfaction, and demographic characteristics [5].

The first formal theory of voluntary fluctuation was described by March and Simon in 1958 when they made a paradigm shift that existed at that time. From that point on, and until 1970, was a period referred to as the foundational models period. The period around the 1980s was the time of theory testing and served as an introduction for models that appeared in the 1990s and became widespread after that. The early 21st century was marked by meta-analyses and the creation of new constructs through the concept of turnover intention [5]. It is extremely important to note that models and theories related to turnover intention are frequently referenced and identified in the literature. In her paper, Ngo-Hena states that models are closely related to theories, and that the difference between theories and models is not always clear [6]. Peterson and Bredow clearly distinguish between models and theories. The main components of theories are concepts and propositions, and core knowledge is considered an essential feature of any profession. These include models, definitions, constructs, and analyses where the interrelationship between the observed constructs and the accompanying variables used to explain the occurrence of certain phenomena is crucial. On the other hand, conceptual models are used to define the purpose and objective in building theoretical frameworks [7].

### 1.2. Conceptual Models of Turnover Intention

Turnover intention is considered in the literature as a complex concept, because it is associated with economic, organisational, and psychological outcomes that depend on a variety of factors [8] and have implications for organisations and individuals alike [9], and that cannot be measured with a single variable. A concept represents a different perception that varies from one individual to another and refers to interrelated ideas that represent and carry a mental image of a phenomenon. Concepts are focused on a specific phenomenon; they are more abstract and less explicit and specific than theories. They develop through three stages: formulation, modelling, and validation. They provide a solid background for building relevant theories, and researchers use them as a guide for developing research ideas [7]. Throughout history, numerous turnover intention models have been presented and analysed. The most far-reaching models that had the most far-reaching impact on the development of the concept of turnover intention and theoretical frameworks are presented in the continuation.

The first published formal theory about turnover intention initiated an entire conceptual era. In 1977, Mobley and Price were among the first researchers who tested various turnover intention models, which were based on the Theory of Organisational Equilibrium. They identified a more comprehensive turnover process and outlined the sequence of steps that employees go through before making the actual decision to quit [10]. Their intermediate linkages model proposed a set of realizations in the process of withdrawal (e.g., thoughts of abandonment, expected benefits) and job search behaviour (e.g., evaluation of alternatives) that associate job dissatisfaction with actual withdrawal. In particular, Mobley presented job dissatisfaction as the main construct and showed that its effects that lead people to think about leaving their jobs. It is interesting to note that the aforementioned authors were among the first to also identify the potentially mitigating effects on turnover intention [6,11].

Based on Price’s earlier work, Price and Mueller developed a causal turnover model in 1981 that identified the antecedents of job satisfaction and turnover intention, and added organisational commitment as a mediator between these two variables [12]. Other signs of turnover intention were, among other things, the nature of the job (e.g., a routine job), involvement, job commitment, and family connections. Price’s work represented a significant horizontal and vertical shift in the turnover model development by introducing job satisfaction [6,13]. Characteristic of the research of these authors was the specificity of the sample, since the participants were exclusively RNs. The research showed that studies based on specific groups of subjects were extremely important for the development of theoretical frameworks [6].

An interesting approach to turnover intention was also presented by Hulin and Hanisch in 1991 in their general withdrawal model. The model is based on the presumption that job dissatisfaction in general or some specific aspects trigger a set of behavioural and cognitive responses. Psychological and behavioural withdrawal are only a part of the comprehensive set of adaptive behaviours, which also include modified behaviour and attempts to influence better work outcomes. Actual turnover intention is merely a subset within the withdrawal construct, which also includes alternative actions, such as tardiness, absenteeism, and retirement [13,14]. In 1994, Lee and Mitchell presented the unfolding model, in which they showed that turnover decision is not always the result of accumulated job dissatisfaction, but can occur even without much reflection. They suggested several decision-making paths that employees can take before actually deciding to quit their job [10]. In general, their model highlights the complexity and dynamics of the turnover process and suggests that future researchers who wish to study turnover intention should consider the ways in which people leave their jobs [13]. This paper outlines the complex process by which employees, as individuals, evaluate their feelings, personal situation, work environment, and ultimately make the decision over time to stay with or leave the organisation. They noted that existing models of employee turnover are too simplistic, and that turnover intention can develop in many ways. In addition, one of the key events that trigger turnover is system shock, described as an event that causes individuals to evaluate their current and potentially future jobs. They also emphasized the urgent need for a new theory of turnover intention that systematically analyses all the important facts that have taken place in the last 50 years [10].

In 2002, Steel presented an analysis that identified two other important reasons for employees’ turnover in situations where they do not have alternative employment. The first was having an alternative source of income and not being forced to work, and the second was receiving a spontaneous job offer [15].

In 2004, Maertz and Campion identified the proximal causes of turnover intent and, consequently, the best predictors of turnover intention behaviour. They combined content and process models of turnover intention and showed that the motivational forces of commitment and withdrawal are systematically related to the type of turnover decision [16]. This suggests that different groups of employees are motivated by different triggers. It also suggests that these causes play a role in the effects of all other major constructs in the literature. A key finding of the empirical study was that employees who quit without a job alternative had stronger negative affect than those who chose otherwise, indicating the importance of the effect of impulsive quitting [13,16]. Using theoretical approaches, researchers have developed several empirical models that interpret individuals’ behaviour. Common themes among these models point to the fact that behaviour in turnover intention is a multifaceted process that includes attitudes, decisions, and behavioural components. The more recently proposed models are often continuations or fine-tunings of earlier models that are considered to be the basis of the current concept of turnover intention and related theories [6].

Despite numerous studies, many questions remain unanswered about the mechanisms that drive nurses to quit and the causes and consequences that this phenomenon entails at the personal, organisational, and social levels [17].

### 1.3. Constructs

Based on previous studies, researchers have used numerous constructs to measure, explain, and describe turnover intention. Most commonly, they analysed job satisfaction, stress, emotional exhaustion, income, working conditions, autonomy, recognition, and respect within a team of health professionals, personal characteristics, leadership skills, work environment, and loyalty to the organisation [18,19,20,21]. These constructs are presented in continuation of the subsections: work motivation (Section 1.3.1), nursing practice environment (Section 1.3.2), job satisfaction (Section 1.3.3), and absenteeism (Section 1.3.4).

#### 1.3.1. Work Motivation

The individual’s motivation is deeply rooted in the Self-Determination Theory—SDT [22,23]. It is a widely accepted theory applied in various fields (e.g., education, healthcare, sports) as well as in organisational management [24]. Central to SDT is the difference between autonomous motivation and controlled motivation. Controlled motivation is influenced by extrinsic regulation (social and material) and introjected regulation, whereas autonomous motivation is influenced by identified regulation and intrinsic goals [25]. It is interesting to note that professional motivation in nursing has not been analysed as a separate factor, but instead it has been analysed through or within other factors. Turnover intention, older age, and low motivation of the newly employed are listed in the literature as reasons for the current nurse shortage [26].

#### 1.3.2. Nursing Practice Environment

The nurses’ working environment is a complex construct to conceptualise and measure. Its theoretical underpinnings span into organisational, occupational, and labour sociology, and it is defined as a set of organisational determinants in the work environment that impact professional nursing practice [27]. Emphasis was placed on more intense research in nurses’ work environment to better understand of the turnover intention [28].

#### 1.3.3. Job Satisfaction

Job satisfaction is defined as an individual’s complex perception that includes certain assumptions and beliefs about a particular job (cognitive component), feelings toward the job (affective component), and job evaluation (evaluative component) [29]. Nurses’ job satisfaction can be divided into three categories: those related to the organisation and performance of the job, those related to interpersonal relationships, and those related to the personal characteristics of the employees themselves [30].

#### 1.3.4. Absenteeism

Absenteeism is defined as the employee’s intentional or regular absence from their workplace [31]. It is also defined as absence from work in the sense of a shortage of workforce due to the inability of employees to perform efficiently, for personal, legal, or illness-related reasons. Sick leave-related absenteeism is defined as the altered perception, ability, and motivation of an employee, usually due to illness or injury. This leads to staff shortages and consequently jeopardises the efficiency and quality of care while increasing the burden on other employees [32].

Based on our previous work demonstrating the content and construct validity of variables of individual constructs [33], which was a pre-test to the current study, we decided to select, examine, and present those that have either a positive or negative significant effect on turnover intention, though this time on a larger number of subjects, as proposed in the previous study. In addition, the purpose of this study was to present a conceptual model based on the effect of the predictors that influence nurses’ turnover intention.

## 2. Materials and Methods

### 2.1. Study Design and Participants

A quantitative, non-experimental research design was used. An anonymous survey was conducted. A closed-ended questionnaire was used as an instrument. The questionnaire was sent via email using the web-based survey tool 1KA [34]. Data were collected between December 2019 and December 2021 at the University of Rijeka, Faculty of Health Studies (Croatia), where 308 registered nurses (RNs) voluntarily responded to the questionnaire. The study included fully employed RNs with high school vocational education and training (VET) and Bachelor of Science (BSc) nurses who are continuously and directly employed in healthcare. To obtain a heterogeneous sample, we collected data from nurses from all three levels of health as well as social welfare institutions from different Croatian counties. The binary regression sample size formula *n* = 100 + 50i [35] was used to determine the sample size of the study, where i represents the number of predictors. The binary regression analysis with the highest number of predictors, i.e., predicting job satisfaction, was used as a reference. In the pre-test for the current study [33], a total of i = 4 predictors were found to be significant, resulting in a minimum sample size of 300 participants.

### 2.2. Instrument

The questionnaire was based on the Multidimensional Work Motivation Scale (MWMS) and the Practice Environment Scale of the Nursing Work Index (PES-NWI), as well as a section with closed-ended questions to collect demographic data (e.g., age, gender, years of service as an RN), employment information, absenteeism (number of days absent in the past 12 months), and a stand-alone question that measured the level of job satisfaction. The MWMS was developed for the application of SDT in practice [23], while the PES-NWI [36] was designed for the analysis of nurses’ work environment. MWMS consists of six constructs: amotivation, external regulation (social), external regulation (material), introjected regulation, identified regulation, and intrinsic motivation, while PES-NWI of five: nursing foundations for quality of care; nurse participation in hospital affairs; nurse manager ability, leadership, and support of nurses; staffing and resource adequacy; and collegial nurse-physician relations. PES-NWI was developed based on the characteristics of hospitals known to attract employees (so-called “magnet hospitals”). The validity and reliability of the Croatian version of both instruments has been confirmed in previous studies [33,37]. Permission to use and adapt both instruments was obtained from the authors of both questionnaires by e-mail.

The level of job satisfaction was measured in accordance with the global approach on a scale of 1–10, where 1 represents dissatisfaction and 10 complete satisfaction. The global approach is based on the definition that job satisfaction is a general affective attitude towards one’s job and organisation. It can be understood as a one-dimensional construct, and it is measured by a single-level scale, i.e., based on the answer to a completely unambiguous question: “How satisfied are you with your job?” [38]. This was later recoded into a dichotomous variable: low job satisfaction (for median and below) and high job satisfaction (above median).

Turnover intention was measured with a dichotomous (yes/no) variable: “Have you considered changing your job in the last year?” Absenteeism was measured based on days of absence from work during the previous year.

### 2.3. Ethical Considerations

All participants were informed of the research objectives and asked to participate voluntarily in the study. They had the right to withdraw from the study at any time without any consequences. The study was conducted in accordance with ethical principles and human rights standards [39]. Ethical approval was granted by the Ethical Committee of the Faculty of Health Studies in Rijeka and the Faculty of Medicine in Osijek (both in Croatia, EU). Confidentiality of participants was ensured both during and after the study.

### 2.4. Data Analysis

Statistical analysis was performed using MedCalc19.5.1 (MedCalc Software Ltd., Ostend, Belgium) and IBM SPSS Statistics 24 (IBM Corp., Armonk, NY, USA) with a selected significance level of α = 0.05. All *p*-values were two-sided.

All categorical data were represented by absolute and relative frequencies, whereas numerical data were represented by the arithmetic mean and standard deviation or the median and interquartile range if the data were not normally distributed. The Shapiro–Wilk test was used to check the normality of the distribution [40].

Comparison of categorical data within groups and between groups was performed using the Chi square test. Correlations between numerical variables were tested using the non-parametric Spearman’s rank correlation test (r_s_). The Mann–Whitney U test was used to test the differences in numerical variables between two groups. Binary regression analysis was used to investigate the predictors of turnover intention. All the assumptions regarding the use of this statistical analysis (e.g., sample size, multicollinearity) were carefully considered [41].

## 3. Results

A total of 308 participants were included in this study, with a median age of 30 years. They were predominantly female, RN with high school vocational education and training (VET) (Table 1). Two significant predictors of turnover intention were identified when binary regression was applied: job satisfaction as a negative predictor with OR = 0.23 and absenteeism with OR = 2.5 (Table 2). Furthermore, four significant predictors of job satisfaction were identified: two negative predictors amotivation with OR = 0.59 and identified regulation with OR = 0.54 and two positive predictors intrinsic motivation with OR = 1.67 and nurse manager ability, leadership, and support of nurses with OR = 1.51 (Table 3).

In the preliminary analyses conducted to investigate multicollinearity between potential predictors, the examined variable years of work experience was excluded from further analysis because it was strongly correlated with age (r_s_ = 0.930, *p* < 0.001), with no statistically significant differences in participants with high and low job satisfaction (Mann–Whitney U test, *p* = 0.550).

**Figure 1:**
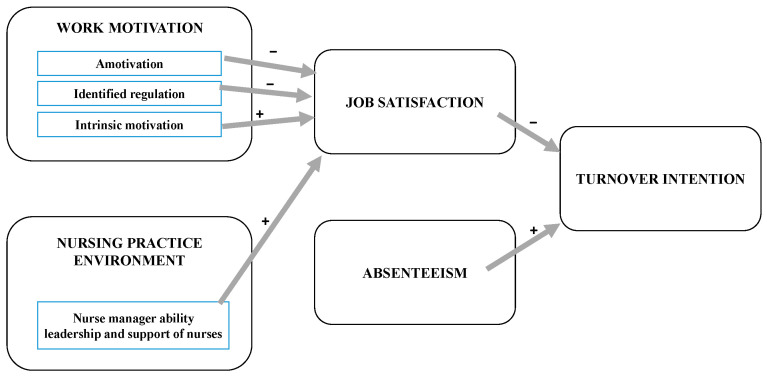
Conceptual model based on the impact of predictors that influence nurses’ turnover intention.

The first binary regression model significantly predicted turnover intention (χ^2^ = 52.148, d.f. = 4; *n* = 256; *p* < 0.001; Nagelkerke R^2^ = 28.0%), while the second binary regression model significantly predicted job satisfaction (χ^2^ = 61.640, d.f. = 14; *n* = 254; *p* < 0.001; Nagelkerke R^2^ = 29.0%). The attempt to identify predictors of absenteeism was unsuccessful as the binary regression model was not significant (χ^2^ = 11.854, d.f. = 14; *n* = 254; *p* = 0.62; Nagelkerke R^2^ = 5.5%). The conceptual model of turnover intention was constructed based on binary regression results and is presented in Figure 1.

## 4. Discussion

Any progress towards a better understanding of the RN turnover intention is of utmost importance. The World Health Organisation predicts a shortage of 15 million health workers by 2030 [42]. This will result in the largest expected skills shortage, which could trigger global competition for qualified health workers. We also need to highlight work motivation as one of the important factors in solving the recruitment and retention problems in the healthcare sector.

Middle-income countries will face labour shortages, as their demand for labour will exceed supply [43]. The economic impact of nurses leaving the healthcare system cannot be fully represented due to a lack of consistent definitions and measurements. However, it is estimated that the cost is four to five times higher as productivity decreases with new hires [44]. When employees leave their jobs, valuable financial and social capital is lost, affecting the morale of other employees and the reputation of the organisation, as well affecting teamwork and work processes [1].

In 1981, Price and Mueller tested their prevailing conceptual model exclusively on RNs to explain turnover intention. The relatively rapid recognition and persistent use of their model compared to those of other authors contributed to its legitimacy and subsequently triggered a series of studies on the causes of turnover intention among nurses [13,45]. Many authors describe organisational and individual factors that influence job satisfaction, turnover intention, and actual quitting. Lake, in her study, integrated specific variables that pertain to the nursing profession (burnout and autonomy) [46]. Brewer–Kovner emphasises the economic factor by including a robust set of variables (labour market demand and household demands) to predict factors influencing the turnover of young RNs [18]. Inspired by the development of various models throughout history, in this study, we have examined the relationship between the predictors that influence nurses’ turnover intention and have presented a conceptual model based on the results.

Many studies on work motivation are based on the examination of intrinsic motivation, therefore the application of SDT in RN work motivation contributes to a better understanding of the concept of motivation [47]. Identifying factors that influence the nurses’ motivation is considered a preventive tool in dissatisfaction and turnover intention [48].

Amotivation is described as the absence of motivation for an activity [23]. In the present study, amotivation has a significant negative impact on the level of job satisfaction. Furthermore, the amotivation is emphasized as a negative predictor of job performance because it has to do with not being motivated at all [49]. This is not surprising, because, without motivation, no one will enjoy working, because motivation is the drive that makes you work towards a goal.

Our results show that intrinsic motivation had a significant positive impact, while the identified regulation had a negative impact on the level of job satisfaction. In the literature, intrinsic motivation is described as doing an activity for its own sake, i.e., because it is intrinsically interesting and enjoyable, while identified regulation refers to doing an activity because one identifies with its meaning/value and voluntarily accepts it as one’s own [23].

An intrinsically motivated worker does not work just to earn money to satisfy his/her needs or those of his/her family but works because of the satisfaction he/she receives from the challenges of work. This provides the opportunity to use his/her knowledge, skills, and potential, thereby developing a sense of accomplishment and self-fulfilment, which in turn makes him/her marketable in society [49]. Intrinsic motivation factors of nurses’ job satisfaction have a significant impact on the performance, stability, and productivity of the health institution [50]. Nurses’ lifelong learning has a significant impact on performance and productivity [51]. Accordingly, continuing professional development (CPD) programmes are central to nurses’ lifelong learning and are an important aspect that keeps nurses’ knowledge and skills up to date. This requires different learning methods and ways of acquiring and building knowledge. To achieve this, nurses can take different approaches to acquire knowledge: CPD, through formal learning, courses, or workshops, as well as informal learning in the workplace, self-reflection, review of literature for best evidence through journal clubs, and mutual feedback. Evidence from CPD literature indicates that many nurses prefer informal learning methods in the workplace and find that the most meaningful learning occurs in interaction with their colleagues. Nurses were found to consider informal learning methods such as supervision, participation in team meetings/briefings, and mentoring. Organisational culture played an important role in staff professional development [50,51]. The main challenge for nursing is capitalising on the workplace as a learning resource that can integrate learning with development, improvement, knowledge translation, inquiry, and innovation. This requires skilled facilitators, particularly for systems’ leaders [52].

In previous studies, identified regulation as part of autonomous motivation has been associated with increased job satisfaction [25,53]. The result presents a paradoxical situation, because Croatian nurses who identify with their vocation and find meaning in their job and internalise the values that the work of a nurse entails are likely to feel less satisfied in their job [33]. If a worker identifies with and/or genuinely enjoys their work, motivation by earnings may be secondary [54].

Of all the SDT motivation types in this study, only intrinsic motivation has a positive impact on Croatian nurses’ job satisfaction. RNs tend to be more motivated by intrinsic motivation factors than other occupational groups. Intrinsic motivation factors have a significant impact on nurses’ performance; it is a form of natural motivation that can increase the RNs’ interest in performing their tasks. A 2019 study by Gunawan et al. states that hospital management needs to create and improve work motivation that can affect the RNs’ performance [55]. If managers know what motivates staff, it can have an impact on performance results. This is not surprising as, Maslach and Leiter [56], in their six areas of the work–life model, emphasised the importance of intrinsic motivation through intrinsic rewards such as recognition for one’s contributions at work. Managers are advised to identify the needs of nurses and design a relevant motivational program to encourage nurses to achieve maximum performance [55]. Similar to our results, Moll-Khosrawi et al., in 2021, also found that job satisfaction was positively correlated with intrinsic motivation and negatively correlated with amotivation [57].

Our results suggest that, of all the PES-NWI constructs, only nurse manager ability, leadership, and support of nurses was found to be a valid predictor of job satisfaction. In the literature, the construct nurse manager ability, leadership, and support of nurses refers to the ability of head nurses to be good nursing managers and leaders, to support other nurses, and to have their backs when making decisions [36]. Maslach and Leiter [56] also emphasize the importance of managers who can improve intrinsic motivation through intrinsic rewards. This suggests that a nurses’ manager can have a significant impact on employees’ intrinsic motivation. The nurse manager is responsible for a large area of a healthcare organisation and manages large budgets and a large number of RNs. Therefore, an RN should not be promoted to the role of nurse manager without the necessary training [58]. Both nurses who wish to be promoted to nurse manager and current nurse managers should attend training programs. They also need to develop advanced management skills to meet current and future challenges [59]. The healthcare industry, in particular, generally suffers the consequences of absenteeism and turnover and has one of the highest turnover rates of any industry. Supervisors or managers with strong leadership and motivational skills are essential to achieving the desired behaviours and attitudes in employees. Nurse managers with higher emotional intelligence have a greater potential to be successful in a leadership role. Emotional intelligence can be developed and trained through nurses’ lifelong learning. Their skills affect the nurse’s behaviour, which ultimately affects their leadership skills, such as decision-making, performance, and productivity [60].

Previous research has shown that collegial support is a factor that can help reduce turnover among staff [42]. A study conducted in the Netherlands shows that the organisational or managerial interventions have the greatest impact in preventing increased turnover [61].

Several authors have shown that personal characteristics such as age [4,62,63] and years of work experience [4] are associated with job satisfaction. No similar results were found in our study.

### 4.1. Implications for Nursing Practice

Knowing the intrinsic and extrinsic motivators that drive people to engage in nursing is an important aspect of recruitment. Our plan is to obtain additional data on RN turnover at the national level to better understand this phenomenon and contribute with our research to the development of a new approach in the recruitment process. Given the current RNs’ work environment, the challenge for healthcare management is to find out how to improve motivational scores through their work. Although there is empirical evidence for some of the constructs in this study, much more attention should be given to research aimed at understanding the nurses’ turnover intention in terms of individual behaviour. More conceptual models should be developed with the antecedents and consequences of turnover intention to better understand this phenomenon. As mentioned above, there is a need to implement targeted lifelong training programmes of nurses—managers with the aim of enhancing and improving the necessary competencies for leadership and the management of material and human resources (e.g., Emotionally Intelligent Leadership, or transformational leadership program). Emotional intelligence is an essential attribute of an effective leader because it affects job satisfaction, atmosphere, and the way people work. For nurse managers, emotionally intelligent leadership is thought to potentially impact staff retention, teamwork, quality of patient care, and job satisfaction. Leadership style impacts nurse training and support, improving the work atmosphere, reducing nurse mental health problems, and staff retention [64].

Transformational leadership is a type of leadership style characterised by a leaders’ ability to understand their organisation culture and reimagine and rebuild it according to a new vision. This authentic form of leadership embraces innovation and creativity while requiring competence in building trust and relationships and rational compassion [65].

We would like to encourage Croatian researchers and all relevant stakeholders to participate in national/EU/global initiatives on nursing education and retention.

### 4.2. Limitations of the Study

There are two limitations to this study that should be considered before generalising the results of this study. First, the results are based on data collected using a web-based questionnaire. Although the survey was anonymous, participants may not have provided entirely truthful responses. This raises several issues, as such that the observation of participants may lead to bias in their actual responses. Therefore, it remains a challenge to choose research methods and data collection procedures that overcome these issues. Second, the study was conducted in one country. Hence, further replications should be performed in various settings to confirm the validity of the presented conceptual model. Authors should discuss the results and how they can be interpreted from the perspective of previous studies and of the working hypotheses. The findings and their implications should be discussed in the broadest context possible. Future research directions may also be highlighted. We point to four directions for future research: (1) conducting an in-depth study of nurses who recently left their previous jobs and identifying the actual reasons for turnover (e.g., accepting a job abroad, low salary) and identifying and classifying their new job positions, (2) examining the impact of employee turnover on other team members and the organisation as a whole, (3) investigating whether nurse managers have acquired adequate leadership skills and competencies, and (4) better clarifying the impact of absenteeism as a predictor of turnover intention.

## 5. Conclusions

The presented conceptual model indicates the strategic issues that need to be addressed to retain the nursing workforce. Intrinsic motivation had a significant positive impact, while identified regulation had a negative impact on the level of job satisfaction. Nurse manager ability, leadership, and the support of nurses were found to be valid predictors of job satisfaction. There is a need to ensure positive perceptions and support from managers, maintain intrinsic motivation, and promote higher levels of motivation to achieve a satisfactory level of job satisfaction. Organisations need to ensure that managers and supervisors are properly trained to adequately support their employees.

## Figures and Tables

**Table 1:** Participants’ sociodemographic characteristics.

Variable	Values	*n* (%)	*p* *
Gender	Male	54 (17.5)	*<0.001*
	Female	254 (82.5)	
Marital status	Married	75 (39.3)	0.130
	Civil partnership	52 (27.2)	
	Single	64 (33.5)	
Level of education (*n* = 308)	VET	285 (92.5)	*<0.001*
	BSc	23 (7.5)	
Healthcare level or work institution (*n* = 302)	Primary	93 (30.8)	*<0.001*
	Secondary	75 (24.8)	
	Tertiary	120 (39.7)	
	Educational institution	4 (1.3)	
	Nursing home	5 (1.7)	
	Other	5 (1.7)	
**Variable**	**Median**	**Interquartile range**	**Total range**
Age	30.0	25.0–37.0	20–55
Years of work experience in nursing	10.0	5.0–16.5	0–35
Years of work experience in the current position	5.0	3.0–10.0	0–35
Absenteeism in the past 12 months	3.0	0–14.0	0–365
Overall job satisfaction	7.0	5.0–8.0	1–10

* Statistical significance—Chi-square test.

**Table 2:** Binary regression prediction model for turnover intention.

Variable	β	SE	Odds Ratio	*p*
Amotivation	0.310	0.174	1.363	0.075
External regulation (social)	0.099	0.138	1.104	0.470
External regulation (material)	−0.231	0.140	0.793	0.099
Introjected regulation	0.171	0.098	1.187	0.079
Identified regulation	0.151	0.154	1.163	0.327
Intrinsic motivation	−0.150	0.136	0.861	0.272
Nursing foundations for quality of care	−0.051	0.129	0.950	0.692
Nurse participation in hospital affairs	−0.177	0.127	0.838	0.162
Nurse manager ability, leadership, and support of nurses	0.028	0.129	1.028	0.830
Staffing and resource adequacy	−0.114	0.118	0.892	0.334
Collegial nurse–physician relations	−0.233	0.107	0.792	0.030
Age	−0.003	0.026	0.997	0.895
Gender	−0.182	0.382	0.834	0.635
Years of work experience in the current workplace	−0.020	0.029	0.980	0.481
Job satisfaction	−1.482	0.314	0.227	<0.001
Absenteeism in the past 12 months	0.918	0.324	2.503	0.005
Constant	2.330	1.346	10.278	0.083

**Table 3:** Binary regression prediction model for job satisfaction.

Variable	β	SE	Odds Ratio	*p*
Amotivation	−0.534	0.170	0.586	0.002
External regulation (social)	−0.018	0.133	0.982	0.891
External regulation (material)	−0.013	0.134	0.987	0.920
Introjected regulation	0.049	0.096	1.050	0.609
Identified regulation	−0.614	0.159	0.541	<0.001
Intrinsic motivation	0.514	0.145	1.672	<0.001
Nursing foundations for quality of care	−0.114	0.127	0.892	0.368
Nurse participation in hospital affairs	0.151	0.127	1.163	0.235
Nurse manager ability, leadership, and support of nurses	0.411	0.131	1.508	0.002
Staffing and resource adequacy	0.180	0.115	1.198	0.117
Collegial nurse–physician relations	0.050	0.105	1.051	0.635
Age	0.030	0.024	1.030	0.209
Gender	0.020	0.363	1.020	0.956
Years of work experience in the current workplace	−0.032	0.027	0.969	0.240
Constant	−1.692	1.269	0.184	0.182

## Data Availability

The data presented in this study are available on request from the corresponding author.

## References

[1] Hussein Alkahtani A. (2015). Investigating Factors That Influence Employees’ Turnover Intention: A Review of Existing Empirical Works. Int. J. Bus. Manag..

[2] Ajzen I. (2011). The Theory of Planned Behaviour: Reactions and Reflections. Psychol. Health.

[3] Toode K., Routasalo P., Helminen M., Suominen T. (2015). Hospital Nurses’ Work Motivation. Scand. J. Caring Sci..

[4] Masum A.K.M., Azad M.A.K., Hoque K.E., Beh L.S., Wanke P., Arslan Ö. (2016). Job Satisfaction and Intention to Quit: An Empirical Analysis of Nurses in Turkey. PeerJ.

[5] Hom P.W., Lee T.W., Shaw J.D., Hausknecht J.P. (2017). One Hundred Years of Employee Turnover Theory and Research. J. Appl. Psychol..

[6] Ngo-Henha P.E. (2017). A-Review-of-Existing-Turnover-Intention-Theories. Int. J. Econ. Manag. Eng..

[7] Peterson S.J., Bredow T.S. (2017). Middle Range Theories: Application to Nursing Research and Practice.

[8] Kaur B., Mohindru P.D., Pankaj M. (2013). Antecedents of turnover intentions: A literature review. Glob. J. Manag. Bus. Stud..

[9] Vogelzang C. (2008). The Complexity of Absenteeism and Turnover intention: Direct, Mediation and Moderation Effects. Master’s Thesis.

[10] Lee T.W., Mitchell T.R. (1994). An Alternative Approach: The Unfolding Model of Voluntary Employee Turnover. Acad. Manag. Rev..

[11] Holtom B.C., Mitchell T.R., Lee T.W. (2006). Increasing Human and Social Capital by Applying Job Embeddedness Theory. Organ. Dyn..

[12] Govindaraju N. (2018). Addressing Employee Turnover Problem: A Review of Employee Turnover Core Models. Int. J. Innov. Sci. Res. Technol..

[13] Holtom B.C., Mitchell T.R., Lee T.W., Eberly M.B. (2008). 5 Turnover and Retention Research: A Glance at the Past, a Closer Review of the Present, and a Venture into the Future. Acad. Manag. Ann..

[14] Hanisch K.A., Hulin C.L. (1991). General attitudes and organizational withdrawal: An evaluation of a causal model. J. Vocat. Behav..

[15] Steel R.P. (2002). Turnover Theory at the Empirical Interface: Problems of Fit and Function. Acad. Manag. Rev..

[16] Maertz C.P., Campion M.A. (2004). Profiles in Quitting: Integrating Process and Content Turnover Theory. Acad. Manag. J..

[17] Gilmartin M.J. (2013). Thirty Years of Nursing Turnover Research: Looking Back to Move Forward. Med. Care Res. Rev..

[18] Brewer C.S., Kovner C.T., Greene W., Cheng Y. (2009). Predictors of RNs’ Intent to Work and Work Decisions 1 Year Later in a U.S. National Sample. Int. J. Nurs. Stud..

[19] Goh Y.S., Lopez V. (2016). Acculturation, Quality of Life and Work Environment of International Nurses in a Multi-Cultural Society: A Cross-Sectional, Correlational Study. Appl. Nurs. Res..

[20] Moyce S., Lash R., de Leon Siantz M.L. (2016). Migration Experiences of Foreign Educated Nurses: A Systematic Review of the Literature. J. Transcult. Nurs..

[21] Pélissier C., Charbotel B., Fassier J.B., Fort E., Fontana L. (2018). Nurses’ Occupational and Medical Risks Factors of Leaving the Profession in Nursing Homes. Int. J. Environ. Res. Public. Health.

[22] Gagné M., Parker S.K., Griffin M.A., Dunlop P.D., Knight C., Klonek F.E., Parent-Rocheleau X. (2022). Understanding and shaping the future of work with self-determination theory. Nat. Rev. Psychol..

[23] Gagné M., Forest J., Vansteenkiste M., Crevier-Braud L., van den Broeck A., Aspeli A.K., Bellerose J., Benabou C., Chemolli E., Güntert S.T. (2015). The Multidimensional Work Motivation Scale: Validation Evidence in Seven Languages and Nine Countries. Eur. J. Work Organ. Psychol..

[24] Brenner C.A. (2022). Self-regulated learning, self-determination theory and teacher candidates’ development of competency-based teaching practices. Smart. Learn. Environ..

[25] Gagné M., Deci E.L. (2005). Self-Determination Theory and Work Motivation. J. Organ. Behav..

[26] Niskala J., Kanste O., Tomietto M., Miettunen J., Tuomikoski A.M., Kyngäs H., Mikkonen K. (2020). Interventions to Improve Nurses’ Job Satisfaction: A Systematic Review and Meta-Analysis. J. Adv. Nurs..

[27] Swiger P.A., Patrician P.A., Miltner R.S., Raju D., Breckenridge-Sproat S., Loan L.A. (2017). The Practice Environment Scale of the Nursing Work Index: An updated review and recommendations for use. Int. J. Nurs. Stud..

[28] Hudgins T.A. (2016). Resilience, job satisfaction and anticipated turnover in nurse leaders. J. Nurs. Manag..

[29] Šimunić A., Gregov L. (2012). Conflict between Work and Family Roles and Satisfaction among Nurses in Different Shift Systems in Croatia: A Questionnaire Survey. Arhiv za Higijenu Rada i Toksikologiju.

[30] Barać I., Prlić N., Plužarić J., Farčić N., Kovačević S. (2018). The Mediating Role of Nurses’ Professional Commitment in the Relationship between Core Self-Evaluation and Job Satisfaction. Int. J. Occup. Med. Environ. Health.

[31] Cucchiella F., Gastaldi M., Ranieri L. (2014). Managing Absenteeism in the Workplace: The Case of an Italian Multiutility Company. Procedia Soc. Behav. Sci..

[32] Craft J., Christensen M., Wirihana L., Bakon S., Tsai L., Barr J. (2017). An Integrative Review of Absenteeism in Newly Graduated Nurses. Nurs. Manag..

[33] Smokrović E., Žvanut M.F., Bajan A., Radić R., Žvanut B. (2019). The Effect of Job Satisfaction, Absenteeism, and Personal Motivation on Job Quitting: A Survey of Croatian Nurses. J. East Eur. Manag. Stud..

[34] University of Ljubljana, Faculty of Health Sciences 1KA, Version 22.06.14. https://www.1ka.arnes.si.

[35] Bujang M.A., Sa’at N., Tg Abu Bakar Sidik T.M., Lim C.J. (2018). Sample Size Guidelines for Logistic Regression from Observational Studies with Large Population: Emphasis on the Accuracy Between Statistics and Parameters Based on Real Life Clinical Data. Malays. J. Med. Sci..

[36] Lake E.T. (2002). Development of the Practice Environment Scale of the Nursing Work Index. Res. Nurs. Health.

[37] Smokrović E., Žvanut M.F., Bajan A., Radić R., Žvanut B. (2018). Translation and Validation of the Croatian Version of the Multidimensional Work Motivation Scale. Manag. J. Contemp. Manag. Issues.

[38] Proroković A., Miliša Z., Knez A. (2017). Work Values and Job Satisfaction in View of Certain Sociodemographic Characteristics. Acta Iadertina.

[39] World Medical Association (2013). World Medical Association Declaration of Helsinki: Ethical Principles for Medical Research Involving Human Subjects. JAMA.

[40] Petz B., Kolesarić V., Ivanec D. (2012). Petzova Statistika: Osnovne Statističke Metode Za Nematematičare.

[41] Leech N., Barrett K., Morgan G.A. (2013). SPSS for Intermediate Statistics: Use and Interpretation.

[42] Ahlstedt C., Eriksson Lindvall C., Holmström I.K., Muntlin Å. (2020). Flourishing at Work: Nurses’ Motivation through Daily Communication—An Ethnographic Approach. Nurs. Health Sci..

[43] Liu J.X., Goryakin Y., Maeda A., Bruckner T., Scheffler R. (2017). Global Health Workforce Labor Market Projections for 2030. Hum. Resour. Health.

[44] Bae S. (2022). Noneconomic and economic impacts of nurse turnover in hospitals: A systematic review. Int. Nurs. Rev..

[45] Lake E.T., Sanders J., Duan R., Riman K.A., Schoenauer K.M., Chen Y. (2019). A Meta-Analysis of the Associations between the Nurse Work Environment in Hospitals, and 4 Sets of Outcomes. Med. Care.

[46] Lake E.T. (2007). The Nursing Practice Environment: Measurement and Evidence. Med. Care Res. Rev..

[47] Toode K., Routasalo P., Suominen T. (2011). Work Motivation of Nurses: A Literature Review. Int. J. Nurs. Stud..

[48] Baljoon R., Banjar H., Banakhar M. (2018). Nurses’ Work Motivation and the Factors Affecting It: A Scoping Review. Int. J. Nurs. Clin. Pract..

[49] Apex-Apeh C.O., Ujoatuonu I.V.N., Ugwu J.I., Olowu C.T. (2020). Motivation and Work Environment as Predictors of Job Performance among Nurses. Niger. J. Psychol. Res..

[50] Ayalew E., Workineh Y., Abate A., Zaleke B., Semachew A., Woldegiorgies T. (2021). Intrinsic motivation factors associated with job satisfaction of nurses in three selected public hospitals in Amhara regional state, 2018. IJANS.

[51] Mlambo M., Silén C., McGrath C. (2021). Lifelong learning and nurses’ continuing professional development, a metasynthesis of the literature. BMC Nurs..

[52] Jackson C., Manley K. (2022). Contemporary Challenges of Nursing CPD: Time to change the model to meet citizens’ needs. Nurs. Open.

[53] Van den Broeck A., Howard J.L., Van Vaerenbergh Y., Leroy H., Gagné M. (2021). Beyond intrinsic and extrinsic motivation: A meta-analysis on self-determination theory’s multidimensional conceptualization of work motivation. Organ. Psychol. Rev..

[54] Lam C.F., Gurland S.T. (2008). Self-Determined Work Motivation Predicts Job Outcomes, but What Predicts Self-Determined Work Motivation?. J. Res. Pers..

[55] Gunawan N.P.I.N., Hariyati R.T.S., Gayatri D. (2019). Motivation as a Factor Affecting Nurse Performance in Regional General Hospitals: A Factors Analysis. Enferm. Clin..

[56] Maslach C., Leiter M.P. (2017). New insights into burnout and health care: Strategies for improving civility and alleviating burnout. Med. Teach..

[57] Moll-Khosrawi P., Zimmermann S., Zoellner C., Schulte-Uentrop L. (2021). Understanding Why All Types of Motivation Are Necessary in Advanced Anaesthesiology Training Levels and How They Influence Job Satisfaction: Translation of the Self-Determination Theory to Healthcare. Healthcare.

[58] García A.G., Pinto-Carral A., Villorejo J.S., Marqués-Sánchez P. (2020). Nurse Manager Core Competencies: A Proposal in the Spanish Health System. Int. J. Environ. Res. Public Health.

[59] González-García A., Pinto-Carral A., Pérez-González S., Marqués-Sánchez P. (2021). Nurse Managers’ Competencies: A Scoping Review. J. Nurs. Manag..

[60] Zaki H.N., Abd-Elrhaman E.S.A., Ghoneimy A.G.H. (2019). The Effect of Emotional Intelligence Program on Decision Making Style. Am. J. Nurs. Res..

[61] Van Der Heijden B., Mahoney C.B., Xu Y. (2019). Impact of Job Demands and Resources on Nurses’ Burnout and Occupational Turnover Intention towards an Age-Moderated Mediation Model for the Nursing Profession. Int. J. Environ. Res. Public Health.

[62] Lorber M., Skela-Savič B. (2012). Job Satisfaction of Nurses and Identifying Factors of Job Satisfaction in Slovenian Hospitals. Croat. Med. J..

[63] Wang Y., Dong W., Mauk K., Li P., Wan J., Yang G., Fang L., Huan W., Chen C., Hao M. (2015). Nurses’ Practice Environment and Their Job Satisfaction: A Study on Nurses Caring for Older Adults in Shanghai. PLoS ONE.

[64] Prufeta P. (2017). Emotional Intelligence of Nurse Managers: An Exploratory Study. J. Nurs. Adm..

[65] Hirai Y., Yoshioka S. (2020). Emotional Intelligence and Work Perceptions Among Nurse Managers. Yonago Acta Med..

